# Analyzing the molecular mechanism of lipoprotein localization in *Brucella*

**DOI:** 10.3389/fmicb.2015.01189

**Published:** 2015-10-28

**Authors:** Shivani Goolab, Robyn L. Roth, Henriette van Heerden, Michael C. Crampton

**Affiliations:** ^1^Protein Technologies, Biosciences, Council for Scientific and Industrial ResearchPretoria, South Africa; ^2^Department of Veterinary Tropical Diseases, Faculty of Veterinary Science, University of PretoriaPretoria, South Africa

**Keywords:** *Brucella* vaccine target, lipoprotein localization, *Brucella* lipoprotein, lipoprotein secretion, outer membrane protein, Lol pathway, pathogen-associated molecular patterns, toll-like receptors

## Abstract

Bacterial lipoproteins possess diverse structure and functionality, ranging from bacterial physiology to pathogenic processes. As such many lipoproteins, originating from *Brucella* are exploited as potential vaccines to countermeasure brucellosis infection in the host. These membrane proteins are translocated from the cytoplasm to the cell membrane where they are anchored peripherally by a multifaceted targeting mechanism. Although much research has focused on the identification and classification of *Brucella* lipoproteins and their potential use as vaccine candidates for the treatment of Brucellosis, the underlying route for the translocation of these lipoproteins to the outer surface of the *Brucella* (and other pathogens) outer membrane (OM) remains mostly unknown. This is partly due to the complexity of the organism and evasive tactics used to escape the host immune system, the variation in biological structure and activity of lipoproteins, combined with the complex nature of the translocation machinery. The biosynthetic pathway of *Brucella* lipoproteins involves a distinct secretion system aiding translocation from the cytoplasm, where they are modified by lipidation, sorted by the lipoprotein localization machinery pathway and thereafter equipped for export to the OM. Surface localized lipoproteins in *Brucella* may employ a lipoprotein flippase or the β-barrel assembly complex for translocation. This review provides an overview of the characterized *Brucella* OM proteins that form part of the OM, including a handful of other characterized bacterial lipoproteins and their mechanisms of translocation. Lipoprotein localization pathways in gram negative bacteria will be used as a model to identify gaps in *Brucella* lipoprotein localization and infer a potential pathway. Of particular interest are the dual topology lipoproteins identified in *Escherichia coli* and *Haemophilus influenza*. The localization and topology of these lipoproteins from other gram negative bacteria are well characterized and may be useful to infer a solution to better understand the translocation process in *Brucella*.

## Lipoprotein Localization

Bacteria are classified as gram negative or gram positive based on the structure of the cell wall. Gram negative bacteria have a cell envelope composed of the outer membrane (OM), a thin hydrophilic peptidoglycan layer lining the periplasmic space and the inner membrane (IM). Whereas, the gram positive bacteria cell envelope is composed of a thick hydrophilic peptidoglycan layer and IM. The IM of gram negative bacteria is a phospholipid bilayer while the OM is composed of an asymmetric lipid bilayer with lipopolysaccharides (LPS) orientated toward the outer surface and phospholipids orientated toward the periplasm (Mitchell, 1961; Bladen and Mergenhagen, 1964). Proteins are synthesized in the cytoplasm and may either span the IM with characteristic α-helical stretches causing their retention in the membrane, referred to as Imps or translocate across the IM by means of a signal peptide to form complex β-barrels that span the OM as outer membrane proteins (Omps) (Koebnik et al., 2000). The characteristic β-barrel of Omp is comprised of amphipathic β-strands (with alternating hydrophobic amino acid residues) ensuring their solubility during secretion through the periplasm (Rosenbusch, 1974; Nakamura and Mizushima, 1976; Pugsley, 1993). Lipoproteins are adept at anchoring to either the IM or OM by means of the acyl moieties (hydrophobic interactions) in both gram negative and gram positive bacteria (Braun and Rehn, 1969; Narita, 2011). This review will focus upon lipoproteins in gram negative bacteria, which are exported to the surface of the OM in certain bacterial species. This provides insight to *Brucella* lipoproteins and the reason for their selection as vaccine candidates for treatment against brucellosis.

## The General Secretory Twin-Arginine Translocation Systems: Inner Membrane To Periplasm

In gram negative bacteria, proteins destined for the OM are exported from the IM to the OM via the type I secretion system (T1SS), type III secretion system (T3SS), type IV secretion system (T4SS), or type VI secretion system (T6SS) in a single, direct step. Other proteins, mostly in gram negative bacteria that translocate to the OM, can initially be exported from the IM into the periplasm by means of the general secretory (Sec) or twin-arginine translocation (Tat) systems as protein precursors and thereafter translocated to the OM surface by other systems in a double reaction process. In most gram positive bacteria, translocation of the proteins to the cell surface is simpler and occurs via the Sec or Tat systems, with exception of mycobacteria. Components of the Sec system recognize a hydrophobic N-terminal sequence of the proteins destined for secretion and translocation of the protein occurs in an unfolded state, coupled with ATP hydrolysis and a proton gradient as the energy source (Sjostrom et al., 1987; Bibi, 1998; Papanikou et al., 2007). On the other hand, the Tat system identifies a sequence composed of basic amino acid residues (Ser- Arg- Arg- x- Phe- Leu -Lys) at the N-terminal end of the protein and protein export occurs in a folded state making use of a proton gradient (Pugsley, 1993; Brink et al., 1998; Müller, 2005).

## Protein Secretion Pathways For General Secretory/Twin-Arginine Translocation Systems- Intermediates

Secretion can be further branched to the translocation of Sec/Tat-transported intermediates from the periplasm to external milieu through the chaperone-usher (CU) pilli pathway required for the formation of fimbrial adhesion involved in host attachment and invasion, biofilm formation, cell motility as well as protein and DNA transport export (Hultgren et al., 1991; Hal Jones et al., 1997). The other translocation pathways include the autotransporter (T5SS, type V secretion system); the type II secretion system (T2SS) and the type VII secretion system (T7SS), which form a channel utilized for the translocation of proteins across both the hydrophobic membrane and the cell in gram positive mycobacteria, and lastly the lipoprotein localization machinery (Lol) pathway. No stable intermediates have been identified for T1SS, T3SS, T4SS, and T6SS in the periplasm. Furthermore, the T3SS, T4SS, and T6SS pathways make use of a conducting channel capable of extending from the bacterial membranes to the host cell membrane specifically allowing for the secretion of bacterial virulence factors. Established pathways of lipoprotein secretion from the IM through the OM involve either the T2SS, T5SS, or Lol, or a model employed by bacteria of the class spirochetes referred as the spirochetal model (Pugsley, 1993). The protein secretion pathways extending beyond the scope of this review, i.e., lipoprotein secretion in gram negative bacteria, will not be discussed in further detail.

## Bacterial Lipoproteins Function, Modification, And Localization

Lipoproteins have the ability to localize to diverse regions in the cell. In gram negative bacteria, mature lipoproteins anchor to the IM, the extracellular surface or the peripheral region of the OM, depending upon the distinct function performed in the cell (Wu and Tokunaga, 1986; Narita et al., 2004). Lipoproteins that are anchored peripherally to the OM play an important role in bacterial physiology including envelope stability, cell division, sporulation, conjugation, nutrient acquisition, signal transduction, transport, and protein folding, and in bacterial pathogenic processes for example adhesion, colonization, invasion, and persistence through immune evasion (Mathiopoulos et al., 1991; Sutcliffe and Russell, 1995; Khandavilli et al., 2008; Hutchings et al., 2009; Kovacs-Simon et al., 2011). The specific localization of lipoproteins is dependent on an effective lipoprotein modification and transport system, and a precise lipoprotein sorting mechanism (Hantke and Braun, 1973). In gram positive bacteria lipoproteins are exported through the IM and acylated to ensure secure localization on the bacterial cell surface. However, in gram negative bacteria lipoproteins destined for the OM must seek guidance from a lipoprotein-specific chaperoned pathway (Sutcliffe and Russell, 1995; Narita et al., 2004; Zückert, 2014). It would be naive to mold OM-associated proteins into a specific group due to their diverse nature and function, however, both the β-barrel structure of Omps and lipid modification of lipoproteins are characteristic of OM-associated proteins. Lipoproteins are generally hydrophilic, however, due to the N-terminal lipid moiety are hydrophobic. For this reason the release of lipoproteins does not occur spontaneously and must overcome the energetically unfavorable reaction at which they become detached from the IM, and are transported across the hydrophilic periplasm to reach the OM. The modification of lipoproteins by attachment of the hydrophobic lipid moiety occurs in the hydrophilic periplasm. This alteration does not impede their export from the periplasm to the surface of the OM (Pugsley, 1993; Tokuda and Matsuyama, 2004). Unlike Imps, which share α-helical membrane spanning domains, and integral Omps, which share the characteristic β-barrel domains, lipoproteins are structurally diverse and share only the N-terminal lipid moiety (MacIntyre et al., 1988; Bernstein, 2011; Narita, 2011).

## Lipoproteins And Their “Lipobox”

The first component of lipoprotein secretion translocates preprolipoprotein precursors from the cytoplasm (where they are synthesized) through the IM and into the periplasm via the Sec or Tat systems (Berks et al., 2005; Driessen and Nouwen, 2008; Zückert, 2014). Lipoprotein genes of bacterial origin contain a C-terminal lipobox. It is this four-amino-acid motif, which shapes the molecular platform for *in silico* lipoprotein prediction algorithms (Inouye et al., 1977). With an increase in the number of bacterial genomes sequenced, more lipoproteins continue to be identified resulting in the degeneration of the original lipobox. At present the only conserved residue of the canonical lipobox is cysteine, which forms the target of acylation and is the new N-terminus amino acid (residue at position +1) of the mature lipoprotein (Seydel et al., 1999; Babu et al., 2006; Setubal et al., 2006). This clearly necessitates the development of new algorithms to identify lipoproteins. Following the +1 cysteine, peptide residues comprising the “tether” (second domain) are occasionally disordered, lacking observed secondary structure and vary in terms of length. This “tether” domain not only contains a lipoprotein sorting sequence but connects the lipid anchor to the third domain, thereby positioning lipoproteins in the cell envelope for optimal protein function (Seydel et al., 1999; Schulze and Zückert, 2006; Zückert, 2013, 2014).

## Preprolipoprotein Diacylglyceryl Transferase, Lipoprotein Signal Peptidase, Lipoprotein N’N-acyl Transferase: Lipidation In The Periplasm

**FIGURE 1:**
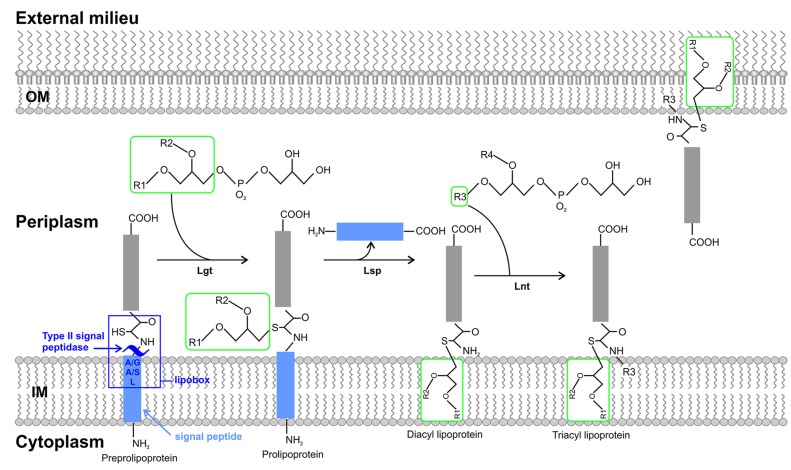
**The biosynthesis and sorting of bacterial lipoproteins.** Prelipoproteins synthesized in the cytoplasm are translocated to the outer surface of the IM by the general secretory (Sec), two-arginine (Tat) pathways. Prelipoproteins possess a consensus motif termed the lipobox, which is a component of the N-terminal signal peptide cleavage site. It is the +1 cysteine residue of the lipobox that undergoes lipid modification in a sequential process catalyzed by three periplasmic enzymes to yield the mature lipoproteins. The first enzyme, preprolipoprotein diacylglyceryl transferase (Lgt) generates a thioether linkage by transferring a diacylglyceryl molecule to the sulfhydryl group of the +1 cysteine. The second enzyme, lipoprotein signal peptidase (Lsp) catalyzes the cleavage of the N-terminal signal peptide at +1 S-diacylglyceryl cysteine. Lastly, lipoprotein N’N-acyl transferase (Lnt) catalyzes aminoacylation of the α-amino group of the S-diacylglyceryl cysteine, producing a mature triacylated lipoprotein. Depending upon the lipoprotein sorting signal the lipoprotein is then targeted for the inner membrane (IM) or the outer membrane (OM). Abbreviations used: inner membrane (IM), general secretory (Sec), two-arginine (Tat), preprolipoprotein diacylglyceryl transferase (Lgt), lipoprotein signal peptidase (Lsp), lipoprotein N’N-acyl transferase (Lnt), outer membrane (OM).

The second component of the biosynthetic pathway of bacterial lipoproteins yields a mature lipidated protein upon post translational modifications of prelipoproteins, catalyzed by three indispensable IM enzymes (**Figure 1**). In detail, preprolipoprotein diacylglyceryl transferase (Lgt) catalyzes diacylation (phosphatidylglycerol attachment to the thiol group) at the conserved cysteine residue of the lipoprotein via a thioester bond. This modification process occurs either within the IM or at the cytoplasmic surface (Tokunaga et al., 1982; Sankaran and Wu, 1994; Selvan and Sankaran, 2008). The signal peptide is then cleaved N-terminally of the +1 cysteine residue by a signal peptidase II enzyme, lipoprotein signal peptidase (Lsp) at the interface of the IM (gram negative bacteria) upon recognition of the lipobox (Tokunaga et al., 1982; Munoa et al., 1991; Vidal-Ingigliardi et al., 2007). The cleavage process avails the N-terminal amine group of the +1 cysteine residue for modification with a third acyl chain via an amide linkage by lipoprotein N’N-acyl transferase (Lnt) thereby completing the membrane anchor in gram negative bacteria (Gupta et al., 1993). This last process of the pathway is expendable in certain gram positive bacteria (Jackowski and Rock, 1986; Vidal-Ingigliardi et al., 2007; Tschumi et al., 2009; Hillmann et al., 2011). IM lipoproteins in gram negative bacteria do not interact with downstream pathways and are retained within the IM. Lipoproteins can localize either to the IM (toward the periplasm) as with gram positive bacteria or the OM as with gram negative bacteria. When translocated to the OM, lipoproteins are anchored to the OM either facing the periplasm, embedded in the inner leaflet of the OM (integral membrane proteins, displaying the C-terminal end to the external surface), anchored to the OM facing the external milieu or cleaved from the OM and released to the external milieu (Pugsley et al., 1986; Schulze and Zückert, 2006; Magnet et al., 2007; Roussel-Jazede et al., 2010; Bernstein, 2011; Zückert, 2014). In gram negative bacteria, OM lipoprotein translocation was first speculated to occur by means of localized fusion of the periplasm (hydrophilic) in between the IM and OM via Bayer’s junctions due to their hydrophobic properties (Bayer, 1968; Bayer, 1991). However, studies conducted by (Tokuda and Matsuyama, 2004), revealed the existence of the Lol pathway. The Lol pathway, T2SS, T5SS, and another pathway well characterized in spirochetes, the spirochetal OM model (involving a specialized flippase complex) are pathways associated with the final process of lipoprotein secretion to the OM (Schulze et al., 2010; Chen and Zuckert, 2011; Chen et al., 2011; Zückert, 2014). These are established pathways, which diversify among the subclasses of Proteobacteria. Examples of various types of surface exposed lipoproteins will be further expounded upon to illustrate these proposed models.

## The Bacterial Lipoprotein Localization Machinery Pathway: Periplasmic Sorting To The Outer Membrane Of Gram Negative Bacteria

The periplasmic sorting pathway exports specific lipoproteins from the IM using chaperones, the ATP binding cassette (ABC) transporter (LolCDE) and energy generated from ATP hydrolysis. A carrier protein (LolA) translocates the lipoproteins through the periplasm, and finally insertion into the inner leaflet of the OM is mediated by an OM receptor (LolB). In *E. coli*, the Lol pathway comprises of LolCDE which is inserted in the IM; LolA, a soluble chaperone located in the periplasm; and LolB, an OM lipoprotein receptor anchored to the inner leaflet of the OM (Matsuyama et al., 1995, 1997; Yakushi et al., 2000). Lipoproteins that are triacylated (i.e., mature and catalyzed by Lnt) are identified by the LolCDE complex. Upon ATP hydrolysis they are transferred to LolA, forming a water soluble complex. Translocation from the periplasm to the OM then occurs by means of diffusion (Tajima et al., 1998; Narita et al., 2002). The lipoprotein-specific periplasmic carrier protein LolA was the first characterized constituent of the Lol pathway and is vital for lipoprotein export through the periplasm. By forming a 1:1 stoichiometric ratio of carrier to lipoprotein, LolA encases the hydrophobic lipid moiety of the lipoprotein (Matsuyama et al., 1995; Tajima et al., 1998). LolB, a lipoprotein itself is situated on the inner periphery of the OM, directing the final step of lipoprotein sorting by accepting lipoproteins from LolA and anchoring them to the OM. As with LolA, knockout gene studies using *lolB* resulted in the accumulation of lipoprotein-LolA complex within the periplasm of *E. coli* (Matsuyama et al., 1997; Tanaka et al., 2001). A high degree of structural homology exists between LolA and LolB based on x-ray crystallography, even though a low degree of identity exists between the primary sequences (Takeda et al., 2003). Both components possess a hydrophobic cavity offering a probable binding site for the acyl chain of the lipoproteins. However, differences in their primary sequences and secondary structure permit the transfer of lipoproteins irreversibly from LolA to LolB in the periplasm (Matsuyama et al., 1997). Hypotheses have been postulated for the interaction of LolA-LolB with lipoproteins. In *Pseudomonas aeruginosa* (γ-Proteobacteria) for example, one acyl chain of the lipoprotein is bound internally, whereas the other two chains are proposed to connect with the proteins using surface hydrophobic patches (Remans et al., 2010). It has been established that homologs for LolB are present β-, γ- and δ-Proteobacteria (absent in α-Proteobacteria). *Brucellae* are placed taxonomically in the α-2 subdivision of the class Proteobacteria, which could suggest that LolA plays a more extensive role in lipoprotein localization. In *Brucella* the mechanism of lipoprotein maturation and translocation has been assumed based on sequence similarity between species (??). Components for lipoprotein maturation (Lgt, Lsp, and Lnt) have been inferred by homology (orthologs closely related species) and components for lipoprotein translocation such as LolA and LolC/E have been predicted (in the absence of proof at protein, transcript, or homology levels), whereas LolD has been inferred by homology (DelVecchio et al., 2002; Halling et al., 2005). The Lol pathway is considered a general mechanism for lipoprotein transport given that the genes encoding Lol proteins are conserved in other gram negative Proteobacteria. It is hypothesized that an Omp or a lipoprotein with a sequence dissimilar to LolB yet still retaining its function could exist, or LolA could be dual functioning, both accepting and incorporating lipoproteins due to the absence of LolB in α-Proteobacteria (Sutcliffe et al., 2012). In *Mycobacterium tuberculosis* for example, a structural LolA/B homolog interacts with all three acyl chains of the lipoprotein. It was assumed that the LolA-B fold is flexible, allowing lipoproteins to encase presenting hydrophobic molecules (Drage et al., 2010).

Studies conducted by deleting LolA allowed for the identification of the LolCDE complex in *E. coli*, which releases lipoproteins into the periplasm. The LolCDE complex is an ABC transporter present in the IM, which initiates the coupling of a lipoprotein to LolA (Yakushi et al., 2000). LolC and LolE are integral Imps homologous to each other, whereas LolD is located within the cytoplasmic leaflet of the IM. LolD possesses ATP-binding domains (conserved Walker A and B motifs) and forms a 2:1:1 stoichiometric ratio with LolC-LolE (Yakushi et al., 1998; Yakushi et al., 2000; Ito et al., 2006). Studies have suggested OM lipoproteins are first retained by LolE causing allosteric modifications increasing LolD’s affinity for ATP. Meanwhile, LolC attaches to LolA and as ATP binds to LolD, lipoprotein interaction with LolE is reduced resulting in lipoprotein relocation to LolA upon ATP hydrolysis and finally the lipoprotein-LolA complex attaches to LolB (Ito et al., 2006; Okuda and Tokuda, 2009; Mizutani et al., 2013; Zückert, 2014).

## Lipoprotein Sorting Within The Periplasm And The Evolution Of The “+2” Rule

The N-terminal end of the mature lipoprotein (triacylated) has a sorting signal, i.e., the suggested lipobox, which is recognized by the Lol pathway for export. In principle, the amino acid residue (i.e., side chain) at position two of the lipoprotein sequence determines membrane localization in *E. coli*. Aspartic acid at +2 functions as an IM retention signal whereas other amino acids at this position function as an OM targeting signal. The aspartic acid functions as a LolCDE avoidance signal for lipoproteins by interfering with the interaction between LolCDE and cysteine (at +1 position) (Yamaguchi et al., 1988; Gennity and Inouye, 1991; Seydel et al., 1999; Terada et al., 2001). Studies involving substituting large non-protein molecules to cysteine at position +2 revealed that LolCDE recognizes the N-acyl S-diacylglyceryl of cysteine and this appears to be the sole structural requirement for LolCDE recognition (Masuda et al., 2002; Hara et al., 2003). Additionally, histidine and lysine at position +3 reduced the stringency of IM retention by the +2 aspartic acid residue. These studies among others rendered the +2 rule a guiding standard instead of a universal rule for lipoprotein sorting by the Lol pathway (Gennity and Inouye, 1991; Zückert, 2014).

## *Brucella* Pathogenesis And Outer Membrane Biogenesis

According to the Food and Agriculture Organization (FAO) and World Health Organization (WHO), brucellosis is one of the most widespread zoonotic diseases in the world (Lopes et al., 2010). The disease is caused by bacteria from the genus *Brucella*. The main economically important species include *B. abortus* (in cattle), *B. melitensis* (in sheep and goats), and *B. suis* (in swine) (Bruce, 1887; Bang, 1897; Traum, 1914). The internalization of *Brucella* within the host cell is a regulated system involving interactions between host cell surface factors and pathogen molecular factors. As part of the intracellular trafficking in host mammalian cells, protein substrates for the T4SS are transported to the host cell cytosol where they allegedly assist translocation and interactions with the endoplasmic reticulum to reach the replicative vacuole. In fact *Brucella* virulence is dependent upon the ability to survive and replicate in the vacuolar phagocytic compartments of macrophages (Arenas et al., 2000; Naroeni et al., 2001). T4SS is categorized as one of the known bacterial secretion systems (discussed previously) and is one of the most important virulence systems identified as crucial in the intracellular processing of *Brucella* within macrophages, which ultimately controls the biogenesis of the intracellular compartment where *Brucella* will finally take residence (Hong et al., 2000; Xiang et al., 2006; de Jong et al., 2008). Similarly, the two-component regulatory system BvrR/BvrS (TCS BvrRS) is another crucial component of the virulence system. The BvrRS is composed of a histidine kinase sensor located in the IM (BvrS) and a cytoplasmic regulator (BvrR). This system controls the expression of a T4SS component, participates in the homeostasis of the OM by controlling the structure of the LPS and the expression of periplasmic proteins and Omps (Omp25 and Omp22) (Guzman-Verri et al., 2002; Manterola et al., 2005; Lamontagne et al., 2007). The interaction between T4SS and BvrRS may epitomize an evolutionarily conserved survival tactic used by the α-Proteobacteria subclass (Comerci et al., 2001; Delrue et al., 2001; Martinez-Nunez et al., 2010). Furthermore, gene expression profiling used to define *Brucella* pathogenesis identified the Omps, lipoproteins, stress response proteins, chaperones, flagellar genes, ABC transport proteins, and genes for LPS and fatty acid biosynthesis as key components for cell envelope or OM biogenesis (Guzman-Verri et al., 2002; Lamontagne et al., 2007; Manterola et al., 2007; Rossetti et al., 2009; Viadas et al., 2009). Immunogenic proteins (particularly Omp25, Omp31, Omp2b) found on the bacterial cell surface are superior vaccine candidates as they establish the initial contact site between the pathogen and the host (Delvecchio et al., 2006). Ultimately the knowledge gained on pathogen-associated factors (with a particular interest to *Brucella* Omps and lipoproteins) have been used for the application of these factors as antigens in vaccine formulation and studies have confirmed their ability to confer protective immunity against *Brucella* and other intracellular pathogens (Fu et al., 2012).

## *Brucella* Outer Membrane Versus Other Gram Negative Bacteria Outer Membrane

What is known about the *Brucella* cell envelope is limited and indirect but does, however, prove the plasticity of its cell envelope (Moriyon and Lopez-Goni, 1998). Subtle yet noticeable differences exist between the cell envelopes of *Brucella* and those of other gram negative Proteobacteria used as model organisms. Studies have verified the failure of using standard bacterial cell fractionation methods for the *Brucella* cell envelope extraction and they also exhibit sensitivity to media classically used in other gram negative bacteria. Interestingly *Brucella* cells cultured in media and within other cells spontaneously release OM components lavish with LPS, native hapten polysaccharide, phosphatidylcholine, Omps, and other proteins maintaining cell viability (Gamazo and Moriyon, 1987). Some of these components inhibit the fusion of phagosomes with lysosomes thereby enabling virulence (Moreno et al., 2004). This shedding is associated with the blebbing of *Brucella* OM and capture of surface and periplasmic constituents (Zhou et al., 1998). The *Brucella* OM tends to form steadier bilayers compared to other gram negative bacteria, owing to the major presence of phosphatidylcholine (lipid) in the OM in contrast to phosphatidylethanolamine in other gram negative bacteria (Moriyon and Lopez-Goni, 1998). The presence of neutral lipids in *Brucella* OM suggests the interaction of positively charged ornithine lipids with the negatively charged phosphates of the lipid A (Martin and Hancock, 1990). The fatty acids in *Brucella* tend to possess longer acyl chains proposing a greater membrane span compared to the model gram negative bacteria (Moriyon and Lopez-Goni, 1998). Contrary to other gram negative bacteria, stronger hydrophobic interactions exist between the *Brucella* Omps and other OM components. The *Brucella* Omps also have a hydrophilic domain enabling interaction with the peptidoglycan layer and surface exposure (Dubray, 1976). However, based on Omp extraction procedures and similarity to *Rhizobium leguminosarum* (a member of the α-2 Proteobacteria subdivision), a portion of Omp could be covalently attached to the peptidoglycan layer (Roest et al., 1995; Vizcaino et al., 1996). Previous studies have shown heat inactivation causes the IM of *Brucella* to collapse, however, the OM maintains morphology, suggesting a greater OM stiffness in *Brucella* compared to other gram negative bacteria (Rubbi, 1991). The anchorage of the lipid (acyl chains) component of *Brucella* lipoproteins is suggested to be stronger too, based on other lipid components in the OM (Moriyon and Lopez-Goni, 1998). Another difference proven is that hydrophobic substrates readily permeate the OM of *Brucella* compared to other gram negative bacteria due to characteristics of the LPS (Douglas et al., 1984; Martinez de Tejada and Moriyon, 1993). Moreover, resistance to the bactericidal oxygen-independent systems of phagocytes characterizes the *Brucella* OM as a result of the resistance the LPS have to polycations (Martin and Hancock, 1990; Martinez de Tejada et al., 1995). The characteristics of the *Brucella* cell envelope define its pathogenicity and resistance. These findings will provide crucial insight into developing an understanding of *Brucella* lipoproteins localization within the *Brucella* OM given their (*Brucella* vs. other gram negative bacteria) variation in OM components.

## Outer Membrane Proteins Accommodated In The *Brucella* Outer Membrane

The *Brucella* cell envelope is constituted of an IM, periplasm (peptidoglycan with soluble components) and OM like other gram negative bacteria (Dubray, 1976; Moriyon and Lopez-Goni, 1998). The OM of *Brucella* is riddled with Omps (comparable with other gram negative bacteria), which were first isolated during the extraction of the cell envelope in the 1980s. These Omps were categorized based upon their molecular mass into group 1 (94 or 88 kDa), group 2 (36–38 kDa), and group 3 (31–34 and 25–27 kDa) with several of them forming tight complex connections with the LPS and peptidoglycan layer (Winter, 1987). Group 2 Omp were classified as porins and lipoproteins bearing similarity to *E. coli* Braun’s lipoprotein (Lpp) (Douglas et al., 1984). Further classification of the group 2 (Omp2a and Omp2b) and group 3 proteins (Omp25 for 25–27 kDa and Omp31 for the 31–34 kDa) designated them as major Omps (Verstreate et al., 1982). Studies have confirmed the surface exposure of the major Omps based upon experiments with the use of monoclonal antibodies (MAbs) (Cloeckaert et al., 1990, 1991; Bowden et al., 1995). The ability of anti-Omp MAbs to bind to the OM surface of *Brucella* Rough (R) and Smooth (S) strains was assessed using ELISA. Labeling with MAb specific for the *Brucella* Omps revealed these Omps have epitopes that are exposed to the OM surface in S*-Brucella* cells. Moreover, this exposure is greater in R*-Brucella* and varies at species level. In order to understand the situation in the OM of the S-*Brucella* cells, it is thus suggested that the LPS with shorter O-chains are favored spatially relative to certain Omps, and/or the native hapten polysaccharides are irregularly dispersed on the OM surface (Gómez-Miguel et al., 1987; Cloeckaert et al., 1990; Bowden et al., 1995). Previous studies performed *in vitro* proposed the ability of antibodies to assist macrophage (cell mediated immune response) opsonization of *Brucella* (Ralston and Elberg, 1969). In mice, high protection was conferred by polyclonal immune sera that was raised against the peptidoglycan layer (reacted with Omps and S-LPS using immunoblots). However, protection conferred by the anti-Omp MAbs was lower in comparison to that conferred by the anti-S-LPS MAbs. Lower protection could be correlated to weaker opsonization as a result of low antigen-antibody accessibility and avidity (Cloeckaert et al., 1991). These investigations formed the basis of using *Brucella* Omps to further probe the effect these Omps would have on *Brucella* virulence.

## *Brucella* Porins (Group 2 Outer Membrane Proteins)

Omp2a forms a monomeric pore in the OM, while Omp2b forms a trimeric channel composed of oligomers (in the presence of SDS, it is resistant to heat denaturation), both typical of some bacterial porins (Marquis and Ficht, 1993). Structural differences exist between the two Omps but do not differentiate which is more efficient or demonstrates better activity (Cloeckaert et al., 2002). However, the expression of Omp2a during intracellular growth of *Brucella* species has not been confirmed to date. Secondary structure prediction methods revealed both these porins have a 16 stranded β-barrel domain with eight large surface loop regions that are exposed to the external milieu (Mobasheri et al., 1997; Paquet et al., 2001). Major differences in the porins are two insertions and/or deletions which exist at the surface exposed loop 3 and loop 5, which vary between the *Brucella* species. It is suggested that pore constriction of *Brucella* porins is most likely exerted by loop 5, instead of the loop 3 (Paquet et al., 2001). Loop 6, loop 7, and loop 8 were identified as epitopes based on analysis with other chimeric porins and studies conducted with MAbs indicated a strong surface exposure of these loops, which is in agreement with the proposed role in bacterial adhesion of the Arg- Gly- Asp (RGD)-containing L6 loop (Cloeckaert et al., 1990; Campbell et al., 1994; Bogdan and Apicella, 1995; Bowden et al., 1995).

## *Brucella* Omp25/Omp31 Family Similarity And Differences

At the level of DNA, a high degree of similarity is observed among the *Brucella* species. However, differences in pathogenicity and host preference that are noted could relate to OM composition. The Omp25/Omp31 family of *Brucella* is composed of seven homologous Omps (Omp22, Omp25b, Omp25c, Omp25d, Omp31b, Omp31, and Omp25). Some of these Omps may be involved in virulence with the encoding genes displaying DNA polymorphisms (Edmonds et al., 2002; Salhi et al., 2003; Vizcaíno et al., 2004; Martín-Martín et al., 2008). It was speculated that *Brucella* Omp25 shares identity with *E. coli* OmpA and even though they are not homologous and differ in size, it is suggested that these β-barrel proteins play a similar function in the OM (Moriyon and Lopez-Goni, 1998). Topology predictions suggest Omp25 is composed of eight stranded transmembrane β domains with large surface-exposed loops. A variation observed in the binding patterns of anti-Omp25 MAbs to *B. ovis* Omp25 suggests an antigenic shift exists due to a 36 bp deletion localized to the last surface-exposed loop at the C-terminal end (Cloeckaert et al., 2002). Like *E. coli* OmpA, *Brucella* Omp25 binds detergents and LPS, causing a characteristic (of β sheets) heat-modifiable electrophoretic migration (Moriyon and Berman, 1983; Moriyon and Lopez-Goni, 1998).

Interspecies differences are described for Omp31. Omp31 is absent in *B. abortus* and a nine nucleotide substitution is observed between *B. ovis* in comparison to *B. melitensis*. This substitution results in a difference in antigenicity between *B. ovis* and *B. melitensis* (Vizcaino et al., 2001b; Cloeckaert et al., 2002). Structural differences in the processing of Omps between gram negative bacteria are evident; an example is the heterologous expression of *B. melitensis* Omp31 in *E. coli.* In contrast to *B. melitensis*, in *E. coli* Omp31 does not seem to interact with the peptidoglycan. This suggests that the interaction of the peptidoglycan to the OM allows a greater “OM stiffness” in *Brucella* compared to other gram negative bacteria (Vizcaino et al., 1996). In other *Brucella* species Omp31 forms oligomers, which are resistant to denaturation by sodium dodecyl sulfate at low temperatures and as such is classified as a porin (since it displays porin characteristics) in *Brucella* species with lower *omp2* gene expression (i.e., Omp31 compensates for Omp2). Functional studies are required to explain these porin characteristics of *Brucella* Omp since in *E. coli* porins are homologous, but *Brucella* Omp2b and Omp31 are divergent (Cloeckaert et al., 1996; Moriyon and Lopez-Goni, 1998). If porin activity occurs as a result of Omp31, its absence in *B. abortus* should have an effect on cell viability. This function could be compensated for by a similar protein, which may suggest the high level of Omp2b in *B. abortus* (Salhi et al., 2003; Caro-Hernandez et al., 2007; Martín-Martín et al., 2009*)*. Based on topology predictions, Omp31, like Omp25, possesses an eight stranded β-barrel domain, however, with much larger surface exposed loops. Three amino acid residue substitutions form part of an immunodominant region on the N-terminal end of surface exposed loop 1 explain the perceived antigenic variation between Omp31 in *B. melitensis* and *B. ovis* (Vizcaino et al., 2001a,b). Furthermore, Omp25 and Omp31 of *Brucella* reveal a significant level of identity to Omps of other members of α-2 Proteobacteria including *Rhizobium, Sinorhizobium, Mesorhizobium, Agrobacterium, Bartonella, and Rickettsia* species (Moreno and Moriyon, 2002). Also, even though Omp31 is a major Omp, its absence has a compensatory effect on the Omp25/31 family that does not influence virulence. Omp31b shares 61% identity with Omp31, however, is absent in *B. melitensis* but identified in *B. abortus* and *B. suis* (yet to be identified in *B. ovis*) using antibody reactivity and protein detection techniques (Salhi et al., 2003; Martín-Martín et al., 2009). Mutant BvrRS *Brucella* strains are avirulent in the mouse model, which is indicated by decreased cell invasion, inhibition of intracellular replication and lysosome fusion and the inability to replicate intracellularly by *Brucella* (Sola-Landa et al., 1998). Studies involving the disruption of BvrR or BvrS resulted in a reduction of *Brucella* Omp2b, Omp22, Omp25, Omp25c, and Omp31b implicating the Omps in *Brucella* virulence. No reduction in Omp25d was noticeable, suggesting *omp25d* was mostly likely differentially expressed and thus detected at very low levels (Lamontagne et al., 2007). Even though group 3 Omps are suggested as crucial for the integrity of *Brucella* OM owing to their close association with LPS, no fluctuations were observed (Edmonds et al., 2001; López-Goni et al., 2004). The homology shared between the Omp25/Omp31 family could suggest structural and functional interchangeability, maintaining OM integrity and virulence in *Brucella*. Due to this redundancy, investigating single Omp mutants of the Omp25/Omp31 family would be misleading (Salhi et al., 2003; Caro-Hernandez et al., 2007; Martín-Martín et al., 2009). These investigations with the Omp25/Omp31 family and the BvrRS reveal a compensatory mechanism among the *Brucella* species and insight into the combined effort of all OM components of *Brucella* to maintain OM homeostasis, which could be speculated to affect lipoprotein localization.

## Lipoproteins Accommodated In The *Brucella* Outer Membrane

The *B. abortus* genome contains approximately 80 genes encoding putative lipoproteins (Chain et al., 2005). Nonetheless, Omp10, Omp16, Omp19, and Omp89 are the four minor Omps previously identified by the use of MAbs that have been examined in much greater detail compared to the other putative lipoproteins (Cloeckaert et al., 1990). Three of the four minor Omps (Omp19, Omp16.5, and Omp10) are low molecular weight proteins, display poor polymorphisms and are expressed in all six *Brucella* species including their biovars (Thirkell et al., 1991; Tibor et al., 1996; Vizcaíno et al., 2000). Based on their amino acid sequences, they are mainly hydrophilic and display similarity to the peptidoglycan-associated lipoproteins (Pal) of other gram negative bacteria hence their association to the peptidoglycan (Moriyon and Lopez-Goni, 1998). Like *E. coli* Lpp, they are lipoproteins (however, Omp89 is not characterized as a lipoprotein) predicted to have three fatty acids connected to an N-terminus glycerylcysteine and the N-terminal signal peptides contain a tetrapeptide with high similarity to the consensus sequence, i.e., the lipobox (Tibor et al., 1999). It is through these lipid moieties that they are periplasmically linked to the OM and it is through these longer acyl chains that these lipoproteins are more strongly anchored to the *Brucella* OM in comparison to other Pal of gram negative bacteria (Moriyon and Lopez-Goni, 1998). Studies performed revealed a similar pathogenic mechanism was observed in macrophages when the lipidated Omp19 (but not the unlipidated form or LPSs) stimulated dendritic cell maturation thereby producing a Th1 type immune response (Giambartolomei et al., 2004; Zwerdling et al., 2008). It is this lipid moiety that is shared by all bacterial lipoproteins that display the same ability to down-regulate antigen capture and increase T-cell response (Chain et al., 2005; Zwerdling et al., 2008). This will be discussed further in the review with regards to Toll-like receptors (TLRs).

## *Brucella* Phenotypic Studies Using Phagocytic Cells To Determine The Effect Of Lipoprotein Gene Mutations

The characteristic and hence pathogenic function of *Brucella* lipoproteins has been assessed by introducing mutations in the genes to determine the resultant phenotype. The model system used to assess the effect of attenuation caused by *Brucella* mutant strains is growth within professional and non-professional phagocytic cells (bovine macrophage cells and human epithelial cells), where its replicative niche is established (Stabel and Stabel, 1995; Delrue et al., 2001). Virulent *Brucella* fail to fuse with lysosomes; by contrast avirulent *Brucella* co-localize with the endosome post-infection (Arenas et al., 2000). By mimicking *in vitro* host intracellular defences, such as treatment with a polycationic peptide (polymyxin B) or with a reactive oxidative agent (H_2_O_2_), resistance conferred can be extrapolated to the characteristic phenotype of the *Brucella* cell envelope (Martinez de Tejada et al., 1995; Freer et al., 1996; Sola-Landa et al., 1998). Furthermore, virulence in mice, sensitivity to bovine complement-mediated lysis (extracellular killing) and growth in minimal media should be assessed in conjunction with *in vitro* studies (Corbeil et al., 1988). Deletions of the immunoreactive lipoproteins, *Brucella omp19* and *omp10* genes were assessed previously (Tibor et al., 2002). The *omp10* mutant resulted in decreased survival rates in mice, and defective growth in media. However, neither *in vitro* growth nor OM characteristics were compromised. Thus it is suggested that replication is impeded in phagocytes during the intracellular stage with the *omp10* mutant. With the *omp19* mutant no splenomegaly (enlarged spleen, clinical symptom) was observed in mice, a lower growth rate was observed *in vitro* suggesting sensitivity to conditions within phagosomes before growth in an intracellular replication compartment. An increase in sensitivity to bovine serum complement was also observed for the mutant (Tibor et al., 2002). Additionally, *omp19* deletion in the R-*Brucella* strain did not impact the survival of mice (Vemulapalli et al., 2000). These observations indirectly suggest Omp19 maintains interactions between OM components and hence the LPS lattice, thereby affecting the extracellular and intracellular survival of *Brucella* (Freer et al., 1996). *Brucella* lipoproteins continue to be identified and similar results were obtained with a conserved 20 kDa lipoprotein mutant (Kim et al., 2013). Mutations were introduced using a transposon mutagenesis screen (Kim et al., 2012). The *Brucella* mutant strain depicted a lower rate of intracellular replication in comparison to the wild type strain in human epithelial cells and macrophages. Moreover, fewer mutants were recovered from the spleen of infected mice compared to infection with the wild type strain (Kim et al., 2013). These experiments provide evidence on the possible use of *Brucella* lipoproteins as candidate vaccines; however, results in the natural host would be crucial. Studies involving the disruption of BvrR or BvrS resulted in a substantial increase in periplasmic proteins, chaperones, and transporters (for, e.g., the inorganic ABC transporter family) with a reduction in Omp25/Omp31 family. The unexpected increase occurred as a result of nutritional stress displaying no auxotrophic defects. A substantial increase in Omp19 was observed. However, Omp10 was mostly likely differentially expressed at levels too low to be detected (Lamontagne et al., 2007). This implies that *Brucella* virulence should be examined as a multidimensional component where compounds play a coordinated role to adapt to various environments.

## The Topology Of *Brucella* Lipoproteins In The Outer Membrane

The Omp10, Omp19, and Omp16 *Brucella* lipoproteins were also suggested to be localized to both sides of the membrane based on antibody accessibility studies (Gómez-Miguel et al., 1987; Cloeckaert et al., 1990; Bowden et al., 1995). This unifies strongly with the existence of dual topology proteins (Lpp, Pal, and P6) identified in other gram negative bacteria and will be expounded on further (Bernstein, 2011; Cowles et al., 2011; Michel et al., 2013, 2015). Omp16 is homologous to the Pals of other gram negative bacteria, however, no homology has been found for Omp10 and Omp19 with other bacteria (Tibor et al., 1994, 1996). *Brucella* Omp10, Omp16, and Omp19 satisfy the characteristics of lipoproteins. Studies using an inhibitor of signal peptidase II resulted in the accumulation of the lipoprotein precursors and immunoprecipitation studies using anti-Omp MAbs confirm the lipid modification of these minor Omp. These studies have provided insight on a proposed topology model for Omp10 and Omp19 where the lipid moiety attached to the lipoprotein N-terminal end is inserted in the external surface of the OM and these lipoproteins would be localized at interface of the OM and the external milieu (Tibor et al., 1999).

## Dual Topology Proteins In Gram Negative Bacteria

**FIGURE 2:**
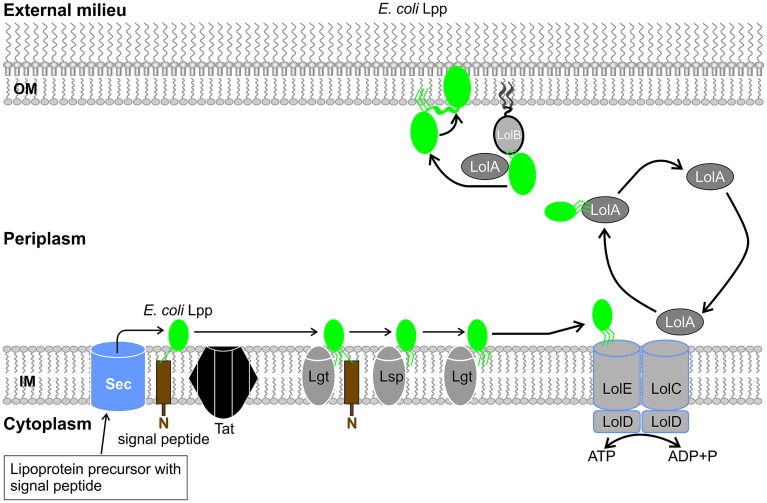
**The proposed translocation pathway for the dual topology *E. coli* Braun’s lipoprotein (Lpp).** After generating the mature Lpp (green structure), the lipoprotein in the inner membrane (IM) interacts with the lipoprotein localization machinery (Lol) located in the periplasm. Adenosine triphosphate (ATP) hydrolysis, an ABC transporter (LolCDE), a carrier protein (LolA) and outer membrane (OM) lipoprotein receptor (LolB) mediates the “mouth-to-mouth” the transfer of Lpp from the IM to the OM. Lpp may reside in the periplasm anchored to the inner leaflet of the OM (bound form) or spans the OM resulting in the surface exposure of its C terminus (free form). Abbreviations used: Braun’s lipoprotein (Lpp), inner membrane (IM), lipoprotein localization machinery (Lol), adenosine triphosphate (ATP), outer membrane (OM), ATP binding cassette (ABC).

It is accepted that *E. coli* Lpp monomers are anchored to the inner surface of the OM (with the N-terminal lipid moiety embedded in the OM) (Yokota et al., 1999). Previous studies suggested that epitopes for both *E. coli* Lpp and Pal are exposed on the surface of *E. coli* (Hellman et al., 1997). These findings were further developed when dual topology characteristics were observed for these *E. coli* lipoproteins. *E. coli* Lpp exists in two distinct forms: a bound form, which resides in the periplasm with its C-terminal covalently attached to the peptidoglycan by transpeptidases, and a free form which spans the OM and is surface-exposed (Magnet et al., 2007; Cowles et al., 2011). **Figure 2** provides insight to a speculated pathway for Lpp translocation in *E. coli*. Approximately 500 000 (of 750 000) copies of Lpp exist in free form in an *E. coli* cell, proving the importance of these structures to the *E. coli* OM bilayer (Braun et al., 1970; Inouye et al., 1972). Crystallographic studies of the soluble recombinant protein resolved a parallel three-stranded coiled-coil structure. The bound form exists as a homotrimer anchored to the OM by its lipid moieties due to the attachment of three lysine residues to the peptidoglycan layer. In comparison, the free form, which also exists as a homotrimer, extends over the OM to form a helix bundle, bearing similarity to transmembrane proteins (Bowie, 1999; Shu et al., 2000).

*H. influenza* P6 is more complex compared to *E. coli* Lpp in terms of structure. It is composed of a mesh of α-helices, loops, and a single β-sheet (Parsons et al., 2006). It is the second lipoprotein (next to *E. coli* Lpp) identified to have dual orientation characteristic in γ-Proteobacteria class of gram negative bacteria. P6 was recognized as a vaccine candidate for the treatment of non-typeable *H. influenzae* infections in humans due to its location on the surface of the OM; the gene is also well conserved among pathogenic strains and was identified as immunogenic (Murphy et al., 1985; Nelson et al., 1988; Bogdan and Apicella, 1995; DeMaria et al., 1996). Since P6 is recognized as one of the Pal, it localizes primarily to the periplasm (Murphy et al., 2006; Shaw et al., 2013; Shaw et al., 2014). This implies that, in contrast to *E. coli* Lpp where more copies of the surface exposed form exist, a greater portion of P6 was exported to the inner periphery of the OM (Michel et al., 2013).

The Lol pathway also exports *E. coli* Pal from the IM into the OM (Liang et al., 2005; Godlewska et al., 2009). The N-terminal end of *E. coli* Pal is suggested to interact with the inner surface of the OM via the lipid moieties and the C-terminal end binds to the peptidoglycan layer, thereby allowing Pal to be located in OM orientated toward the inner surface of the OM (Cascales and Lloubès, 2004; Parsons et al., 2006; Godlewska et al., 2009). The *E. coli* Pal lipoprotein shares similarity in terms of sequence and structure to P6, which would suggest that the lipoproteins possibly share function, protein interaction, and hence localization (Parsons et al., 2006; Godlewska et al., 2009). Pal is localized like P6 mainly to the periplasm, associated with the C-terminal domains of OmpA, TolB (periplasmic protein), TolA, and the peptidoglycan layer (Clavel et al., 1998; Godlewska et al., 2009). *E. coli* Pal and *H. influenza* P6 both form a monomeric α/β sandwich with the secondary structures (α-helices, loops, and a β-sheet) and have a high (50%) sequence identity (Chen and Henning, 1987; Parsons et al., 2006). Recently evidence of a dual topology role of Pal was established (Michel et al., 2015). Approximately 25% of the lipoprotein is exposed to the OM surface (similar to *H. influenza* P6). The dual orientation was further implicated in a pathological mechanism since the surface-exposed Pal may be released from the OM during sepsis (Hellman et al., 2000).

## *Brucella* Lipoproteins And Their Similarity To Other Gram Negative Dual Topology Proteins

Due to the similarity of *Brucella* Omp16 with other Pal of gram negative bacteria, this may imply that *Brucella* Omp16 may be composed of a complex folding involving α/β secondary structure and like *H. influenza* P6 it may have surface exposed regions or be completely exposed to the external milieu. Even though the idea of a dual orientation lipoprotein might be probable, immunological studies have not confirmed the existence thereof. Previous studies in other gram negative bacteria have proposed the dual orientation of the lipoproteins takes place as a result of gene duplication followed by divergent evolution of topology; this may also occur post protein synthesis in the endoplasmic reticulum (Rapp et al., 2006). The dual orientation of membrane proteins has been conserved through evolution signifying an importance for the double orientation of these proteins (Michel et al., 2013). It is believed that bacterial lipoproteins have a common post-translational modification pathway (Pugsley, 1993). Based on the successful modification of *Brucella* Omp10, Omp16, and Omp19 when expressed in *E. coli*, it can be assumed the pathway for lipoprotein maturation is functionally conserved between *Brucella*, *E. coli* and other related gram negative bacteria (Tibor et al., 1999). The integration of lipoproteins into the OM could indicate the existence of novel types of membrane channels. A translocase (or a folding catalyst) reserved for interconverting the two forms has been implicated (Cowles et al., 2011).

## Dual Topology *Brucella* Lipoproteins And Using Experimental Models Described For *E. coli* Of Lol And Other Pathways

Surface proteolysis is most commonly used to validate the surface exposure of a protein experimentally yet some of these gram negative bacteria proteins are protease resistant. *Brucella* Omps are no exception. Group 3 Omps form tight interactions to LPS, conferring resistance of these Omps to protease digestion (Gamazo and Moriyon, 1987). Recently dual topology proteins were identified using a novel, protease independent method which involved labeling with the water-soluble biotin reagents succinimidyl-6′-(biotinamido)-6-hexanamido hexanoate (NHS-LC-LC-biotin) and sulfosuccinimidyl-2-(biotinamido)ethyl-1,3-dithiopropinate (Sulfo-NHS-SS-biotin), followed by cell disruption techniques to quantify the population of cell surface Omps versus periplasmic Omps. These compounds are impermeable to the *E. coli* OM due to their relative high molecular mass and hydrophobicity and therefore surface exposed *E. coli* Omps can be labeled (Cowles et al., 2011; Michel et al., 2015). In assessing the dual topology of *Brucella* lipoproteins on the *Brucella* OM this would be impractical since these biotin analogs establish hydrophobic aliphatic biotin modifications on the proteins. This may alter the characteristics of surface exposed Omps (in particular lipoproteins as they are regarded as hydrophilic with the hydrophobic moiety embedded in the OM) in *Brucella* since hydrophobic compounds spontaneously permeate the OM of *Brucella* (Douglas et al., 1984; Martinez de Tejada and Moriyon, 1993). Another labeling reagent, which could be of use in the *Brucella* OM, is polyethylene glycol (PEG)-NHS-biotin. This analog is a primary-amine reactive, due to the PEG backbone it is hydrophilic and its high molecular mass makes it inaccessible to the periplasm (Nakae and Nikaido, 1975; Decad and Nikaido, 1976). Alternately, indirect visualization of surface-exposed lipoproteins using confocal microscopy could allow detection using a MAb specific for the lipoprotein and a secondary antibody conjugated fluorophore. As a negative control, a lipoprotein mutant strain may be used (Michel et al., 2015).

## *Brucella* Lipoproteins And The Lipoprotein Localization Machinery

### Outer Membrane Lipoprotein Carrier, LolA and LolB?

**FIGURE 3:**
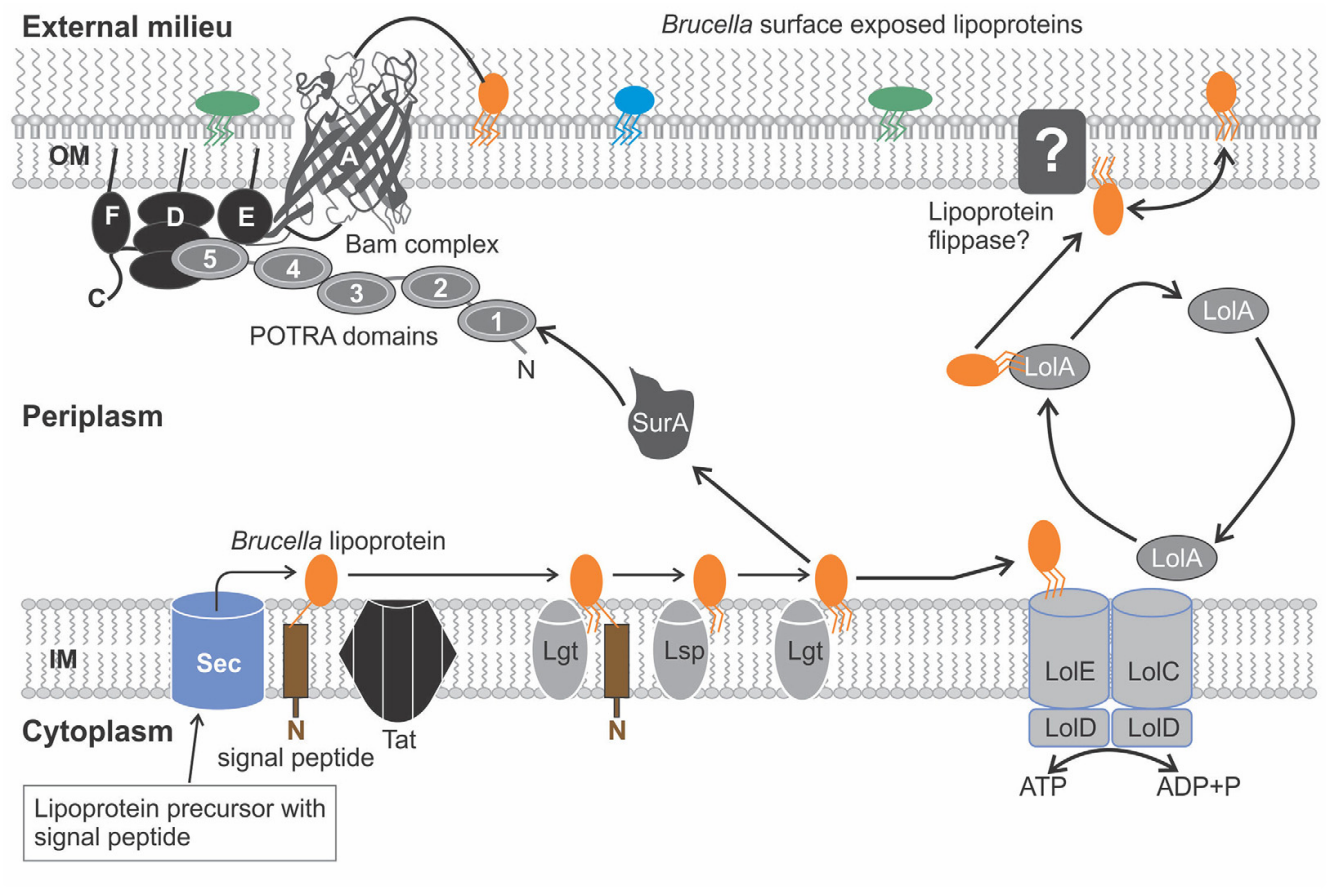
**The translocation pathways for the surface exposure of lipoproteins in *Brucella*.** Prelipoproteins synthesized in the cytoplasm undergo lipid modification generating a mature, triacylated lipoprotein. *Brucella* lipoproteins (orange, green, and blue structures) may be released from the inner membrane (IM) to the outer membrane (OM) using the lipoprotein localization machinery (Lol) pathway, which is propelled by Adenosine triphosphate (ATP hydrolysis), an ATP binding cassette (ABC) transporter (LolCDE) and a carrier protein (LolA). LolB is absent in α-Proteobacteria, as such it is proposed lipoprotein flippase located in the OM transfers lipoproteins to the OM. Another proposed translocation pathway involves the export of the *Brucella* lipoproteins through the β- barrel domain of β- barrel assembly complex (Bam) complex embedded in the OM, assisted by the periplasmic chaperones (SurA) to the surface of the OM. The *Brucella* Bam complex lacks the BamB and BamC components found in other gram negative bacteria, however, a BamF component with a conserved sequence motif related to the BamC component may perform a similar role. Abbreviations used: inner membrane (IM), outer membrane (OM), lipoprotein localization machinery (Lol), adenosine triphosphate (ATP), ATP binding cassette (ABC), β- barrel assembly complex (Bam).

*Brucella* lipoproteins like those present in other gram negative bacteria are transported into the periplasm via the Sec or Tat pathways. They then undergo the process of lipidation catalyzed by the Imps, Lgt, Lsp, and Lnt (**Figure 1**). Depending on the function of the specific *Brucella* lipoprotein, the lipoprotein (like in other gram negative bacteria) is secreted to the OM (**Figure 3**). Components of the Lol pathway required for the modification and translocation of *Brucella* lipoproteins have been characterized (??). All components, except for LolB have been identified in *Brucella* since this gene is absent in α-Proteobacteria indicating the presence of a “dual functioning LolA” or another Lol component dissimilar to the conventional LolB as previously suggested (Narita, 2011; Sutcliffe et al., 2012). In other gram negative bacteria LolB is structurally similar to LolA and also possesses a hydrophobic cavity; however, LolB has a greater affinity for the lipid moiety due to the predominant presence of smaller hydrophobic amino acid residues such as leucine and isoleucine as opposed to bulky aromatic residues present in LolA. An efficient one-way lipoprotein transfer in the periplasm occurs as a result of the formation of a hydrophobic channel between LolA and LolB and the difference in affinity for lipoproteins, since energy sources are absent in the periplasm (Narita, 2011). If LolA plays a dual functional role in α-Proteobacteria, how does LolA translocate lipoproteins to the OM as ATP hydrolysis only fuels lipoprotein IM detachment and interaction of acyl chain to LolA? Furthermore, LolB is a lipoprotein anchored to the OM by a N-terminal acyl chain and LolA is not. This anchorage was identified as an essential component for incorporation of lipoproteins to the OM. The presence of extra C-terminal loop characteristic of LolA, confirms LolA cannot be targeted to membranes. It was therefore established that although LolA and LolB are structurally similar they are functionally distinct in the sorting of lipoproteins (Okuda et al., 2008; Tsukahara et al., 2009). This evidence suggests the possible existence of another protein dissimilar to LolB in sequence but which would perform the same function as LolB in α-Proteobacteria.

## LolD-LolC/E Lipoprotein Releasing System

In *Brucella*, the lipoprotein releasing system is achieved by a “LolC/E” complex. This substantiates the likelihood of lipoprotein release by LolC–LolD heterodimer since LolE is only present in γ-Proteobacteria and not in the other subdivisions. Conformational changes in LolA are required to accommodate the acyl chains of lipoproteins. This change is achieved by interaction with LolC or LolE; however, LolC and not LolE cross-links with LolA suggesting the transfer of lipoproteins from LolA to LolC. This indicates that LolE is not important for lipoprotein transport in some bacteria since LolC interacts with LolA and would substantiate the absence of LolE in β-, δ-, and α-Proteobacteria (Narita, 2011).

## Establishing The Existence Of The Lol Pathway Components For *Brucella* Lipoprotein Translocation Using Experimental Models

In *E. coli*, lipoprotein components were identified by generating spheroplasts and observing the effect that periplasmic (e.g., LolA) and OM components (e.g., a LolB homolog) have on the translocation of lipoproteins (Matsuyama et al., 1995, 1997; Yokota et al., 1999). Spheroplasts, in which the OM is disturbed, secrete proteins destined for the periplasm or OM. *Brucella* lipoproteins destined for the OM surface would therefore remain intact in the IM, as components for translocation are absent. In order to assess the periplasmic components required for lipoprotein translocation, an isolated periplasmic component (e.g., LolA) combined with the spheroplasts this would result in the secretion of *Brucella* lipoproteins. This periplasmic component would most likely be LolA as identified in other gram negative bacteria (Matsuyama et al., 1995). These secreted *Brucella* lipoproteins form a complex with LolA, which when isolated and incubated separately with the extracted *Brucella* OM component would presumably allow for the translocation and hence incorporation of the *Brucella* lipoproteins in the OM. This would suggest an Omp component is essential for lipoprotein incorporation in the OM. By solubilizing the Omp with detergents and addition of phospholipids a proteoliposome is generated which would determine the OM component required (since LolB is absent in *Brucella*) (Matsuyama et al., 1997; Yokota et al., 1999). Similarly the LolD-LolC/E component located in the IM of *Brucella* may be identified.

The *Brucella* lipoproteins characterized to date have a peripheral location with the anchorage of the lipid moiety to either the outer surface or the periplasmic leaflet of the OM as alluded to with Ab accessibility and similarity with *E. coli* PAL and Lpp. If they are anchored to the periplasmic leaflet, it is likely that the lipoprotein adopts an integral membrane protein conformation resulting in the surface exposure of its C-terminal end (like *E. coli* Lpp). However, it may also be exported to both the inner leaflet and outer leaflet of the OM (like *E. coli* Pal) (Michel et al., 2015). Studies conducted on the orientation of a Pal present in *H. influenza* P6, implies the existence of an energy driven reaction (in order to translocate across the cell envelope) catalyzed by a “flippase” enzyme to chaperone the “flipping” of the lipoprotein to the OM outer surface. The orientation of P6 could possibly occur via the Lol system during or after translocation into the OM (Michel et al., 2013). These suggestions seem most likely to exist for the translocation of *Brucella* lipoproteins since they are similar to these dual topology lipoproteins. In order to identify the OM component (an Omp, or a specific translocase) involved in “flipping” lipoproteins to the surface of the OM, *Brucella* knockout mutants (single mutations) may be created for the OM component. It would be essential to monitor the integrity of the *Brucella* cell envelope (additionally, IM marker detection would be useful). These results may also vary between using the R- and S-*Brucella* strains as previously suggested (Cloeckaert et al., 1990, 1996). Based on these knockout strains, components essential for *Brucella* lipoprotein surface exposure would result in no surface detection using the hydrophilic biotin analog and immunofluorescence techniques described previously (Michel et al., 2015).

## *Brucella* Lipoproteins And The β-Barrel Assembly Complex

The involvement of the Bam (β-assembly machine, consisting of BamA; BamB; BamC; BamD; and BamE) complex (which translocates β-barrel proteins in the OM) could perhaps exist in lipoprotein translocation too (Ruiz et al., 2006; Bernstein, 2011). This was observed for the *Neisseria gonorrhoeae* NalP which is an autotransporter permitting the export of immunogenic proteins into the extracellular milieu by means of its protease domain and can itself be exported from the OM after further N-terminal processing. It is also branded as a lipoprotein with a characteristic C-terminal lipobox within the signal sequence (Van Ulsen et al., 2003). In fact, lipidation was found to be important for NalP’s autotransporter function (Van Ulsen et al., 2003; Roussel-Jazede et al., 2013). The export of NalP in an unfolded state is mediated by the T5SS pathway and includes the Bam complex (lodged in OM) and the integral membrane protein chaperones Skp, SurA, and DegP (located in the periplasm). These chaperones enable NalP’s translocator domain (β-barrel with a narrow hydrophilic pore containing an N-terminal α-helix) to insert into the OM, followed by translocation of its N-terminal passenger domain through the OM and finally its autolytic cleavage. It is suggested that the lipid anchor is incorporated on the OM surface since the N-terminal passenger domain is surface localized; however, the anchor topology is unknown (Oomen et al., 2004; Zückert, 2014). Gram negative bacteria autotransporters are integrated in the OM by a common machinery due to a consensus C-terminal sequence recognized by Omp85, which in turn is responsible for the translocation of other Omp (Robert et al., 2006). In general, three possible post-translocations can occur for the secreted proteins. The protein can remain covalently attached to the translocator domain, can be cleaved yet still remain connected to the OM or, as with NalP, it can be released into the external milieu (Van Ulsen et al., 2003; Zückert, 2014).

Would it be possible for the Bam complex, which mediates translocation of *Brucella* Omps to export *Brucella* lipoproteins resembling a variation of the T5SS model for *Neisseria gonorrhoeae* NalP? Moreover, certain components of the Bam complex are lipoproteins and in *Neisseria meningitidis* for example BamC is regarded a prospective vaccine candidate (surface antigen). The concept of lipoprotein translocation via the Bam complex cannot be considered far-fetched as the functions and interrelation of lipoproteins in the OM of gram negative bacteria are vast (Pajon et al., 2009). Analysis of bacterial genome sequences suggests the OM assembly machinery might be similar in gram negative bacteria (Gatsos et al., 2008). ?? consists of the *Brucella* equivalent components of the β-barrel assembly pathway that are present in other gram negative bacteria and have been demonstrated in *Brucella* based upon sequence similarity. All components and processes are similar in *Brucella* compared to other gram negative bacteria, preceding delivery of protein substrates to the Bam complex (**Figure 3**). SurA is a periplasmic chaperone mediating the translocation of proteins across the periplasm to the OM. In *Brucella* and certain other α-Proteobacteria, SurA is diminished lacking a periplasmic peptidyl-prolyl isomerase domain indicating these domains are not essential for binding protein substrates (Alcock et al., 2008). The main component of the Bam complex, BamA/Omp85 consists of a N-terminal end comprised of multiple polypeptide-transport-associated (POTRA) domains and a C-terminal β-barrel domain embedded in the OM. Structural analysis of the POTRA domains reveal a promiscuous binding capability to diverse peptide substrates. In *E. coli* BamA forms a complex with other lipoproteins namely BamB, BamC, BamD, and BamE. The second, third, and fourth POTRA domains are essential to facilitate interactions with BamB and the fifth POTRA domain with BamD and BamE (Kim et al., 2007; Misra, 2007). Interestingly enough, the *Brucella* BamA homolog possesses all five POTRA domains, although in *Neisseria meningitides* BamA is comprised only of the fifth POTRA domain and maintains its function like the other homologs (Robert et al., 2006; Bos et al., 2007). The lipoprotein components of BamA are assembled and anchored by the lipid moiety in the inner or outer surface of the OM as discussed previously. A BamC homolog could not be inferred in α-Proteobacteria, however, studies conducted using *Caulobacter crescentus* (an α-Proteobacterium) identified a novel BamF component with a conserved sequence motif related to those present in BamC. BamF is found exclusively in α-Proteobacteria, hence also in *Brucella* and is described as having evolved like BamC from the ancestral Bam complex (Anwari et al., 2012). Although BamB is present in some species of α-Proteobacteria, *Brucella* like *Neisseria* lacks a homolog for this component (Bos et al., 2007). It was established that certain protein substrates interact with BamB whereas with others, SurA carrying other protein substrates interacts directly with BamA compensating for the lack of BamB (Vuong et al., 2008). The complete activity and diversity of the Bam complex is largely unknown and could perhaps assist lipoprotein translocation too (Ruiz et al., 2006; Bernstein, 2011).

## *Brucella* Lipoproteins And Other Possible Models

Various studies have established the existence of different models for lipoprotein translocation, which directly relates to the function of the specific lipoprotein. One such example is the pathogenic bacterial species of the spirochete class *Borrelia*, which possess many surface exposed lipoproteins functioning as major antigens, some being vital in the pathogenesis of Lyme disease (zoonotic disease) and relapsing fever. Secretion of the lipoproteins to the external surface is proposed to occur in an unfolded monomeric state (which can occur at the C-terminal end) and once at the surface it assembles into a quaternary structure (Kumru et al., 2011). This development paved the way for another hypothesis on the existence of a periplasmic “holding” chaperone, which interacted with the unfolded lipoproteins as they surface from the IM Sec system into the periplasm. This chaperone then delivers the lipoprotein to a “flippase” complex, which in turn exports it to the OM surface with N-terminal anchored into the OM (Schulze et al., 2010; Chen and Zuckert, 2011; Chen et al., 2011). LolA may function similarly to the “holding” chaperone; however, the involvement of the Lol pathway in this model remains to be resolved (Zückert, 2014). Other studies further implicate the role of the Bam complex in translocation of *Borrelia burgdorferi* lipoproteins (Lenhart and Akins, 2010). The spirochetal model could seem plausible since *Brucella* is absent of LolB, this would suggest the transfer of the mature lipoprotein from LolA to the holding chaperone and “flipping” to the OM surface. Furthermore, the T2SS model has been described for *Caulobacter crescentus* (classified as an α-Proteobacterium) under phosphate starvation conditions (Le Blastier et al., 2010). The T2SS in gram negative bacteria is a double-step mechanism, allowing for the secretion of folded and/or oligomeric proteins to the cell’s external milieu (Filloux et al., 1990). The proteins are translocated across the IM via the Sec or Tat systems thereafter the T2SS secreton, which is composed of three sub-complexes (GspDQ secretin, T2SS proteins and pseudopilus), recognizes and exports the proteins across the OM (Pugsley, 1993). Lipoproteins do not possess a predetermined, universal structure. They vary, from consisting of a large globular domain (*E. coli* BamC) to having a translocator domain (*Neisseria* autotransporter NalP) to a parallel three-stranded coiled-coil structure (*E. coli* Lpp). Barely any detail on the mechanism of lipoprotein translocation to the OM is available due to the variation in biological activity elicited by the lipoproteins and hence the complex machinery associated with lipoprotein transport in these bacteria. However, the mechanisms and intricacies illustrated in other gram negative bacteria have provided a stepping-stone in understanding the translocation of *Brucella* lipoproteins. Different models have been suggested for the translocation of lipoproteins to the OM of gram negative bacteria. These models interlink in mechanism. The Lol pathway could integrate the spirochetal model by use of a “flippase” complex in species devoid of LolB and the spirochetal model may engage LolA of the Lol pathway or a BamA homolog used by the T5SS model. Using these and the T2SS models, a relative understanding and a model for the translocation of *Brucella* lipoproteins will be established. To establish whether the Bam complex or any other model for lipoprotein translocation may potentially be used by *Brucella* instead of the Lol pathway, a knockout mutant *Brucella* strain of all components of the Lol pathway should be created. If lipoprotein translocation and surface exposure occurs without the Lol pathway this would implicate the role of another translocation pathway. The interlinking of various pathways may also be established by the formation of proteoliposomes and including required IM and periplasmic components (for, e.g., LolD–LolC/E and LolA) and OM components (e.g., the Bam complex) for *Brucella* lipoprotein translocation.

## *Brucella* Lipoproteins Alone Are Toll-Like Receptor 2 Agonists As Vaccine Antigens

Pathogens, in particular bacteria, display molecular motifs known as pathogens-associated molecular patterns (PAMPs) that are recognized by the host’s innate immune system, thereby protecting the host from infection. The most common PAMPs are nucleic acids derived from pathogens and molecular components exposed on the surface, which include LPS, peptidoglycan, lipoproteins, and flagellin. Pathogens express a set of PAMPs that interact with multiple host pattern-recognition receptors (PRR). These receptors are grouped into secreted molecules and surface receptors for pathogen engulfment (on phagocytic cells) or the release of cytokines. After the PAMP-PRR interaction, signal transduction causes the activation of transcription factors that ultimately proceeds to the expression of inflammatory cytokines, type I interferon (IFN), chemokines and other compounds requires to elicit an immune response. Bacterial lipoproteins, i.e., *Brucella* Omp16 and Omp19, being lipoproteins are potent activators of the transmembrane proteins, TLRs (a group of PRRs) and in particular the cell surface TLR2, which complexes as a heterodimer with TLR1 or TLR6. Studies were performed using crystal structures of TLR2/1 and TLR2/6 (from model bacteria) heterodimerized with synthetic triacyl and diacyl lipopeptide (modified +1 cysteine residue of the lipoprotein). Crystallography revealed the triacylated lipopeptides/lipoproteins activate via the TLR2/1 heterodimer and diacylated lipopeptides/lipoproteins via the TLR2/6 heterodimer (Takeuchi et al., 2001, 2002; Takeda et al., 2002). Furthermore, it was speculated that the TLR2 bonds with the O-esterified fatty acids, the glyceryl group and the thioether moiety (S-glycerylcysteine residue of triacyl and diacyl lipopeptides) and that the TLR1 uses it hydrophobic cavity to distinguish the amide-linked fatty acid of the triacyl lipopeptide. TLR6, however, does not possess a distinguishing cavity. Other studies have elaborated upon this model suggesting lipidation alone is not solely responsible for TLR2 heterodimer selectivity, but also the amino acid residues after the +1 cysteine (lipidated) residue hence acyl chains position and protein/lipid compositions (Buwitt-Beckmann et al., 2005, 2006; Omueti et al., 2005; Kurokawa et al., 2009). However, TLR2 alone is not involved in controlling *in vivo Brucella* infection. Other PAMPs, such as *B. abortus* DNA was recognized as a TLR9 activator and LPS, and unlipidated Omp16, a TLR4 activator. Furthermore, lipoproteins instead of LPS were identified as the stimulators of the inflammatory response caused by *B. abortus* (Giambartolomei et al., 2004; Kang et al., 2009; Pasquevich et al., 2010; Pasquevich et al., 2011; Delpino et al., 2012; Gomes et al., 2012). Therefore, it is speculated that the use of multiple PAMPs to elicit various components of the immune system should be investigated further in an effort to design an effective vaccine that would target *Brucella* given that the pathogen already establishes a replicative niche within the host. At present the superiority of live-attenuated *B. abortus* vaccines (e.g., S19 and RB51) to stimulate an effective immune response (particularly a T-cell response) supersedes currently developed vaccines against brucellosis, yet at the expense of a constant serological response (Schurig et al., 2002). This review has focused upon experimentally characterized and studied *Brucella* lipoproteins, which have been regarded as protective, surface antigens. However, with the advancement of modern technology such as reverse vaccinology, which allows for the identification of protective antigens that have specific structural, immunogenic and functional qualities based on *in silico* genome analysis, many more OM constituents and their biosynthetic pathways will be documented in the future for the development of a safe and effective *Brucella* vaccine.

## Conflict of Interest Statement

The authors declare that the research was conducted in the absence of any commercial or financial relationships that could be construed as a potential conflict of interest.

## Supplementary Material

The Suplementary Material for this article can be found online at: http://journal.frontiersin.org/article/10.3389/fmicb.2015.01189

## ABBREVIATIONS

*B. melitensis*: *Brucella melitensis*
*B. ovis*: *Brucella ovis*
*B. suis*: *Brucella suis*
CU: chaperone-usher
*E. coli*: *Escherichia coli*
*H. influenza*: *Haemophilus influenza*
IM: inner membrane
Imp: inner membrane protein
Lgt: preprolipoprotein diacylglyceryl transferase
Lsp: lipoprotein signal peptidase
Lnt: lipoprotein N’N-acyl transferase
Lol: lipoprotein localization machinery
Lpp: Braun’s lipoprotein
LPS: lipopolysaccharides
MAb: monoclonal antibodies
OM: outer membrane
Omp: outer membrane protein
PAMPs: pathogens-associated molecular patterns
Pal: peptidoglycan-associated lipoproteins
POTRA: polypeptide-transport-associated
PRR: pattern-recognition receptors
RGD: Arg- Gly- Asp
Sec, system: general secretory
Tat system: twin-arginine translocation
T1SS: type I secretion system
T2SS: type II secretion system
T3SS: type III secretion system
T4SS: type IV secretion system
T5SS: type V secretion system
T6SS: type VI secretion system
T7SS: type VII secretion system
TCS BvrRS: two-component regulatory system BvrR/BvrS
TLRs: toll-like receptors
