# Supplementary material for: Analyzing the molecular mechanism of lipoprotein localization in Brucella
Source: Front Microbiol. 2015 Oct 28;6:1189. doi: 10.3389/fmicb.2015.01189 (PMC4623201; doi:10.3389/fmicb.2015.01189)
Supplement: Supplementary file 1 [file Table_1.DOCX]

***Supplementary Material***

**Analyzing the molecular mechanism of lipoprotein localization in**

***Brucella***

**Shivani Goolab*, Robyn Lindsay Roth, Henriette van Heerden, Michael Craig Crampton**

*** Correspondence:** Shivani Goolab: shivanigoolab@yahoo.com

Table 1: Components of lipoprotein assembly pathway in different *Brucella* species with available information ((DelVecchio et al., 2002; Halling et al., 2005).

| **Bacterial specie** | **Locus Names**  **and database ID** | **Lipoprotein component (length)** | **Localization** | **Sequence similarity** | **Molecular function** | **Protein existence** |
| --- | --- | --- | --- | --- | --- | --- |
| *Brucella abortus*  *Brucella melitensis*  *Brucella suis* | BruAb1_1517 [UniProt:Q57BY9](http://www.uniprot.org/uniprot/Q57BY9)  BMEI0488  UniProt: Q8YIF6  BS1330_I1522  UniProt: Q8FZF5 | Prolipoprotein diacylglyceryl transferase, **Lgt** (281aa) | Cytoplasm (multi-pass membrane protein) | Lgt family | [phosphatidylglycerol-prolipoprotein diacylglyceryl transferase activity](http://www.ebi.ac.uk/QuickGO/GTerm?id=GO:0008961): transfers the N-acyl diglyceride group on what will become the N-terminal cysteine of membrane lipoproteins | Inferred by homology:orthologs exist in closely related species |
| *Brucella abortus*  *Brucella melitensis*  *Brucella suis* | BruAb1_0145 [UniProt:Q57FM7](http://www.uniprot.org/uniprot/Q57FM7)  BMEI1799  UniProt: Q8YES8  BS1330_I0149  UniProt: Q8G308 | lipoprotein signal peptidase, [**LspA**](http://biocyc.org/BABO262698/NEW-IMAGE?type=LOCUS-POSITION&object=GJC2-147&chromosome=NIL) (160aa) | Cytoplasm (multi-pass membrane protein) | [Peptidase A8 family](http://www.uniprot.org/uniprot/?query=family:%22peptidase+A8+family%22) | [aspartic-type endopeptidase activity](http://www.ebi.ac.uk/QuickGO/GTerm?id=GO:0004190): signal peptide cleavage from prolipoprotein | Inferred by homology:orthologs exist in closely related species |
| *Brucella abortus*  *Brucella melitensis*  *Brucella suis* | BruAb1_2131 [UniProt:Q57AA7](http://www.uniprot.org/uniprot/Q57AA7)  BMEI1972  UniProt: Q8YEA6  BS1330_I2152  UniProt: Q8FXT9 | Apolipoprotein N-acyltransferase, **Lnt** (532aa) | Cytoplasm (multi-pass membrane protein) | [CN hydrolase family](http://www.uniprot.org/uniprot/?query=family:%22CN+hydrolase+family%22). [Apolipoprotein N-acyltransferase subfamily](http://www.uniprot.org/uniprot/?query=family:%22CN+hydrolase+family.+Apolipoprotein+N-acyltransferase+subfamily%22) | [N-acyltransferase activity](http://www.ebi.ac.uk/QuickGO/GTerm?id=GO:0016410) transfers the fatty acyl group on membrane lipoprotein and hydrolase activity | Inferred by homology:orthologs exist in closely related species |
| *Brucella abortus*  *Brucella melitensis*  *Brucella suis* | BruAb1_1978  UniProt: Q57AQ2  BAWG_0127  UniProt: D0B3P5  BSUIS_A1843  UniProt: B0CJ05 | Outer membrane lipoprotein carrier protein, **LolA** (230aa) | Peripheral membrane protein | Outer membrane lipoprotein carrier, LolA family | OM lipoprotein carrier LolA-like activity: translocation of lipoproteins from the inner membrane to the outer membrane. | Predicted: without evidence at protein, transcript, or homology levels |
| *Brucella abortus*  *Brucella melitensis*  *Brucella suis* | BruAb1_0838 [UniProt:Q57DS9](http://www.uniprot.org/uniprot/Q57DS9)  BMEI1138  UniProt:Q8YGM0  BS1330_I0820  UniProt: Q8G195 | Lipoprotein-releasing system ATP-binding protein, **LolD** (227aa) | Cell inner membrane; Peripheral membrane protein | ABC transporter superfamilyLipoprotein translocase (TC 3.A.1.125) family | ATP binding,  ATPase activity,  lipoprotein transporter activity | Inferred by homology:orthologs exist in closely related species |
| *Brucella abortus*  *Brucella melitensis*  *Brucella suis* | [BruAb1_0837](http://biocyc.org/BABO262698/NEW-IMAGE?type=LOCUS-POSITION&object=GJC2-849&chromosome=CHROMOSOME-1)  UniProt:Q57DT0  BM28_A0834  UniProt:F2HVM1  BSUIS_A0862  UniProt:B0CLF0 | Lipoprotein releasing system protein,  ABC transporter permease, **LolC/E** (422aa) | Peripheral membrane protein | LolCE; lipoprotein releasing system, transmembrane protein, LolC/E family | [Lipoprotein transporter activity](http://www.ebi.ac.uk/QuickGO/GTerm?id=GO:0042954) | Predicted: without evidence at protein, transcript, or homology levels |
