# Supplementary material for: Analyzing the molecular mechanism of lipoprotein localization in Brucella
Source: Front Microbiol. 2015 Oct 28;6:1189. doi: 10.3389/fmicb.2015.01189 (PMC4623201; doi:10.3389/fmicb.2015.01189)
Supplement: Supplementary file 2 [file Table_2.DOCX]

***Supplementary Material***

**Analyzing the molecular mechanism of lipoprotein localization in**

***Brucella***

**Shivani Goolab*, Robyn Lindsay Roth, Henriette van Heerden, Michael Craig Crampton**

*** Correspondence:** Shivani Goolab: shivanigoolab@yahoo.com

Table 2: Components of outer membrane protein assembly pathway in *Brucella* with available locus database identifications, and other functional information (Anwari et al., 2012; DelVecchio et al., 2002; Gatsos et al., 2008; Halling et al., 2005; Paulsen et al., 2002).

| **Bacterial specie** | **Locus Names**  **and database ID** | **Omp component (length)** | **Localization** | **Sequence similarity** | **Molecular function** | **Protein existence** |
| --- | --- | --- | --- | --- | --- | --- |
| *Brucella abortus*  *Brucella melitensis*  *Brucella suis* | BruAb1_1921 [UniProt: [Q57AV4](http://www.uniprot.org/uniprot/Q57AV4)](http://www.uniprot.org/uniprot/Q57BY9)  BMEI0121  UniProt: [Q8YJG2](http://www.uniprot.org/uniprot/Q8YJG2)  BR1945,  UniProt: [Q8FYD8](http://www.uniprot.org/uniprot/Q8FYD8) | Protein translocase subunit SecA (906aa) | Cytoplasm | [SecA family](http://www.uniprot.org/uniprot/?query=family:%22SecA+family%22) | Component of Sec translocase complex. Interacts with SecYEG preprotein complex.  Couples ATP hydrolysis with protein transport into and across the IM, serving as a receptor for the preprotein-SecB complex  Binds to zinc ions | **Inferred by homology:**orthologs exist in closely related species |
| *Brucella abortus*  *Brucella melitensis*  *Brucella suis* | BruAb1_2047  [UniProt:[P0C125](http://www.uniprot.org/uniprot/P0C125)](http://www.uniprot.org/uniprot/Q57FM7)  BMEI2055  UniProt:[Q8YE23](http://www.uniprot.org/uniprot/Q8YE23)  BR2072  UniProt: [Q8FY19](http://www.uniprot.org/uniprot/Q8FY19) | Protein-export protein **SecB** (163aa) | Cytoplasm | [SecB family](http://www.uniprot.org/uniprot/?query=family:%22SecB+family%22) | Essential for the export of cytoplasmic preproteins. Molecular chaperone, maintaining precursor proteins in a translocation-competent state.  Binds to its receptor SecA | **Inferred by homology:**orthologs exist in closely related species |
| *Brucella abortus*  *Brucella melitensis*  *Brucella suis* | BruAb1_1218 [UniProt: [Q57CS8](http://www.uniprot.org/uniprot/Q57CS8)](http://www.uniprot.org/uniprot/Q57AA7)  BMEI0777  UniProt: [Q8YHM0](http://www.uniprot.org/uniprot/Q8YHM0)  BR1213,  UniProt: [Q8G091](http://www.uniprot.org/uniprot/Q8G091) | **Protein translocase subunit SecY** (446aa) | Cell IM (multi-pass membrane protein) | [SecY/SEC61-alpha family](http://www.uniprot.org/uniprot/?query=family:%22SecY%2FSEC61-alpha+family%22) | Central subunit of heterotrimer SecYEG translocation channel. Predicted to have 10 transmembrane helices with both the N and  C-termini facing the cytoplasm  Forms a lateral gate which opens onto the bilayer is clamped together by SecE. Channel is closed by both a pore ring (SecY) and a short helix plug.  Interacts with the ribosome, SecDF (release protein into perilplasm), and SecA | **Inferred by homology:**orthologs exist in closely related species |
| *Brucella abortus*  *Brucella melitensis*  *Brucella suis* | BruAb1_1254 **UniProt:** [Q57CP2](http://www.uniprot.org/uniprot/Q57CP2)  BMEI0743  **UniProt:** [Q8YHQ3](http://www.uniprot.org/uniprot/Q8YHQ3)  BR1250  **UniProt:** [Q8G063](http://www.uniprot.org/uniprot/Q8G063) | Protein translocase subunit **SecE** (79aa) | Cell IM (single-pass membrane protein) | [SecE/SEC61-gamma family](http://www.uniprot.org/uniprot/?query=family:%22SecE%2FSEC61-gamma+family%22) | Subunit of heterotrimer SecYEG translocation channel. Predicted to have a single transmembrane segment  Clamps together SecY. Possible contact with the channel plug during translocation | **Inferred by homology:**orthologs exist in closely related species |
| *Brucella abortus*  *Brucella melitensis*  *Brucella suis* | BAB1_1160 [UniProt: [Q2YPU5](http://www.uniprot.org/uniprot/Q2YPU5)](http://www.uniprot.org/uniprot/Q57DS9)  BAWG_2818 UniProt: [D0B9B0](http://www.uniprot.org/uniprot/D0B9B0)  BR1137  UniProt: [Q8G0F8](http://www.uniprot.org/uniprot/Q8G0F8) | Preprotein translocase subunit **SecG** (148aa) | Cell IM | none | Subunit of heterotrimer SecYEG translocation channel. Two transmembrane domains (span the IM twice), both of which contribute to the preprotein signal sequences recognition by the translocation complex. The protein also undergoes membrane topology inversion when coupled to the SecA | **Predicted:** without evidence at protein, transcript, or homology levels |
| *Brucella abortus*  *Brucella melitensis*  *Brucella suis* | LepB, BAAA_1000701 UniProt: [C4IPW0](http://www.uniprot.org/uniprot/C4IPW0)  BMEI1288  UniProt: [Q8YG73](http://www.uniprot.org/uniprot/Q8YG73)  **lepB**, BR0660 UniProt: [Q8G1Q1](http://www.uniprot.org/uniprot/Q8G1Q1) | Signal peptidase I (278aa)    Signal peptidase I (260aa) | Cell IM and periplasm | peptidase S26 family | Export β-barrel protein across IM | **Inferred by homology:orthologs exist in closely related species** |
| *Brucella abortus*  *Brucella melitensis*  *Brucella suis* | **lspA**, BruAb1_0145 UniProt:[Q57FM7](http://www.uniprot.org/uniprot/Q57FM7)  **lspA**, BMEI1799 UniProt: [Q8YES8](http://www.uniprot.org/uniprot/Q8YES8)  **lspA**, BR0149 UniProt: [Q8G308](http://www.uniprot.org/uniprot/Q8G308) | Signal peptidase II (160aa) | Cell IM (multi-pass membrane protein) | peptidase A8 family | This protein specifically catalyzes the removal of signal peptides from prolipoproteins | **Inferred by homology:orthologs exist in closely related species** |
| *Brucella abortus*  *Brucella melitensis*  *Brucella suis* | BruAb2_0153 UniProt: [Q579T7](http://www.uniprot.org/uniprot/Q579T7)  BMEII1083 UniProt: [Q8YB18](http://www.uniprot.org/uniprot/Q8YB18)  BRA0157  UniProt: [Q8FXC3](http://www.uniprot.org/uniprot/Q8FXC3) | Seventeen kilodaton protein **Skp** (186aa)  **Skp** (184aa)  **Skp** (190aa) | Periplasm | Skp family | Polypeptide: amino acid ABC transporter substrate-binding protein. Molecular chaperone that maintains the solubility of early folding Omp intermediates during passage through the periplasm. | **Predicted:** without evidence at protein, transcript, or homology levels |
| *Brucella abortus*  *Brucella melitensis*  *Brucella suis* | BAAA_1000726 UniProt: [C4IPY2](http://www.uniprot.org/uniprot/C4IPY2)  BMEI1265 UniProt: [Q8YG95](http://www.uniprot.org/uniprot/Q8YG95)  BR0684 UniProt:[Q8G1M9](http://www.uniprot.org/uniprot/Q8G1M9) | Chaperone **SurA** (318aa)  Survival protein SurA (317aa)  Molecular chaperone SurA (318aa) | Periplasm | peptidase S26 family | Chaperone involved in the correct folding and assembly of Omp. Recognizes specific sequences of aromatic residues and side chain orientation (integral Omp). Possible function for early periplasmic and late OM-associated steps of protein maturation. | **Predicted:** without evidence at protein, transcript, or homology levels |
| *Brucella abortus*  *Brucella melitensis*  *Brucella suis* | BruAb1_1160 UniProt: [Q57CY5](http://www.uniprot.org/uniprot/Q57CY5)  BMEI0830 UniProt:[Q8YHH0](http://www.uniprot.org/uniprot/Q8YHH0)  BR1154  UniProt: [Q8G0E3](http://www.uniprot.org/uniprot/Q8G0E3) | Outer membrane protein assembly factor **BamA/YaeT** (Omp85) (781aa) | Cell OM | [BamA family](http://www.uniprot.org/uniprot/?query=family:%22BamA+family%22) | Part of the outer membrane protein assembly complex, which is involved in assembly and insertion of β-barrel proteins into OM | **Inferred by homology:orthologs exist in closely related species** |
| *Brucella abortus*  *Brucella melitensis*  *Brucella suis* | BAAA_2000354 UniProt: [C4ISA4](http://www.uniprot.org/uniprot/C4ISA4)  BMEI0587  UniProt: [Q8YI58](http://www.uniprot.org/uniprot/Q8YI58)  BR1422  UniProt: [Q8FZQ1](http://www.uniprot.org/uniprot/Q8FZQ1) | Outer membrane protein assembly factor **BamD/YfiO** (323aa)  (309aa)  (287aa) | Cell OM | [BamD family](http://www.uniprot.org/uniprot/?query=family:%22BamD+family%22) | Lipoprotein component involved in assembly and insertion of β-barrel proteins into OM | **Inferred by homology:orthologs exist in closely related species** |
| *Brucella abortus*  *Brucella melitensis*  *Brucella suis* | BAbS19_I07430  UniProt: [B2S520](http://www.uniprot.org/uniprot/B2S520)  BMEI1184  UniProt: [Q8YGH5](http://www.uniprot.org/uniprot/Q8YGH5)  DK68_3136 UniProt: [A0A080PGD7](http://www.uniprot.org/uniprot/A0A080PGD7) | **SmpA/BamE** (167aa)  Small protein a | Cell OM | SmpA / OmlA family protein | Lipoprotein component involved in assembly and insertion of β-barrel proteins into OM | **Predicted:** without evidence at protein, transcript, or homology levels |
| *Brucella* | Unknown | **BamF** | Cell OM | Unknown | Lipoprotein component involved in assembly and insertion of β-barrel proteins into OM. BamC homologues do not exist in α-Proteobacteria. BamF has a conserved sequence motif related to BamC. BamF and BamD can be eluted from the BAM complex under similar conditions as BamC-D component. BamF homologues present in all species of α-Proteobacteria | **Predicted:** without evidence at protein, transcript, or homology levels |
